# Commencement

**Published:** 1879-03

**Authors:** 


### COMMENCEMENT.

The thirty-third annual commencement of the Ohio College of Dental Surgery was held on Wednesday evening, March 6, 1879.

The annual address was delivered by Dr. W. H. Morgan, of Nashville, Tenn., it was replete with good advice and valuable suggestions. The address will appear in the April number of the REGISTER. A response on behalf of the class was given by R. G. Richter, of Milwaukee. The following prizes were awarded, viz: To C. W. Wardle, Cincinnati, of the senior class, a dental engine for the best artificial denture. To R. G. Richter, Milwaukee, one set of instruments, for the best thesis on conseveration of dental pulp. To Arthur G. Rose, Cincinnati, one set of instruments for best essay on pyorrhœa alveolaris. To W. B. Ames, of the junior class, one lathe for the best artificial denture.

The degree of D. D. S. was conferred by the president of the board of trustees, Dr. James Taylor, upon the following gentlemen, viz: W. N. Cameron, Oscar Neise, N. E. Heighlands, W. H. H. Hunter, N. P. Kelly, W. F. Magie, Harry L. Moore, A. G. Rose, J. B. Stevenson, James Stockton, C. W. Wardle, Otto Arnold, Ohio; C. W. Moffett, W. N. Alsop, P. Burgen, W. F. Redman, Kentucky; C. J. Phillips, T. S. Brown, Pennsylvania; W. S. Rawls, E. W. Anderson, Allen Budd, Indiana; R. G. Richter, Wisconsin; J. Sanger, California; J. L. Young, Mississippi.

